# THE ASSOCIATION BETWEEN NEIGHBORHOOD PERCEPTIONS AND RISK OF FALLS

**DOI:** 10.1093/geroni/igae098.1377

**Published:** 2024-12-31

**Authors:** Qun Le, Lingming Chen, Elizabeth Procter-Gray, Meng Zhang, Danielle LoPilato, Annabella Aguirre, Wenjun Li

**Affiliations:** University of Massachusetts Lowell, Lowell, Massachusetts, United States; University of Massachusetts Lowell, Lowell, Massachusetts, United States; University of Massachusetts Lowell, Lowell, Massachusetts, United States; University of Massachusetts Lowell, Lowell, Massachusetts, United States; University of Massachusetts Lowell, Lowell, Massachusetts, United States; University of Massachusetts Lowell, Lowell, Massachusetts, United States; University of Massachusetts Lowell, Lowell, Massachusetts, United States

## Abstract

Fall is a leading cause of injury-related death among old adults. This study is to explore the associations between neighborhood environment perceptions and the risk of falls among old adults. The data was drawn from the Healthy Aging and Neighborhood Study in Central Massachusetts (2018-2024). Data were collected from 379 participants who aged over 65 years and live in rural, suburban, and urban communities. Neighborhood perception was measured using a self-administered questionnaire. Factor analysis was used to derive perception scores in five aspects including neighborhood utilitarian (NU) (range:0-7), sense of community (SEC) (range:0-5), safety of community (SAC) (range:0-8), friendly neighborhood (FN) (range:0-3), and aesthetics of community (AC) (range:0-5). A higher score indicates a more positive perception. The occurrences of falls were recorded using monthly falls calendars. Negative binomial regressions were used to assess the associations of neighborhood perceptions and fall rates adjusting for sociodemographic factors and follow-up time. The participants had high neighborhood perceptions (mean±SD: NU: 2.46±1.69, SEC: 3.34±0.92, SAC: 7.42±0.94, FN: 1.03±0.85, and AC: 3.55±0.86). Lower rates of indoor falls were associated with a higher score in SEC (IRR: 0.8, 95%CI: 0.6-0.9) and SAC (IRR: 0.7, 95%CI: 0.6-0.8). A one-unit higher AC score was associated with 20% lower rates of outdoor falls (IRR: 0.8, 95%CI: 0.6-1.0). Higher scores in the SEC (IRR: 0.8, 95%CI: 0.7-0.9), SAC (IRR: 0.8, 95%CI: 0.7-0.9), and AC (IRR: 0.8, 95%CI: 0.7-1.0) were significantly associated with lower rates of falls in any place. These results suggest the need of considering neighborhood perceptions in fall intervention.

